# Supplementary material for: Longitudinal development of sex differences in the limbic system is associated with age, puberty and mental health
Source: Commun Biol. 2025 Nov 5;8:1524. doi: 10.1038/s42003-025-08866-3 (PMC12589509; doi:10.1038/s42003-025-08866-3)
Supplement: Supplementary file 2 — Supplementary Information [file 42003_2025_8866_MOESM2_ESM.pdf]

Supplementary File

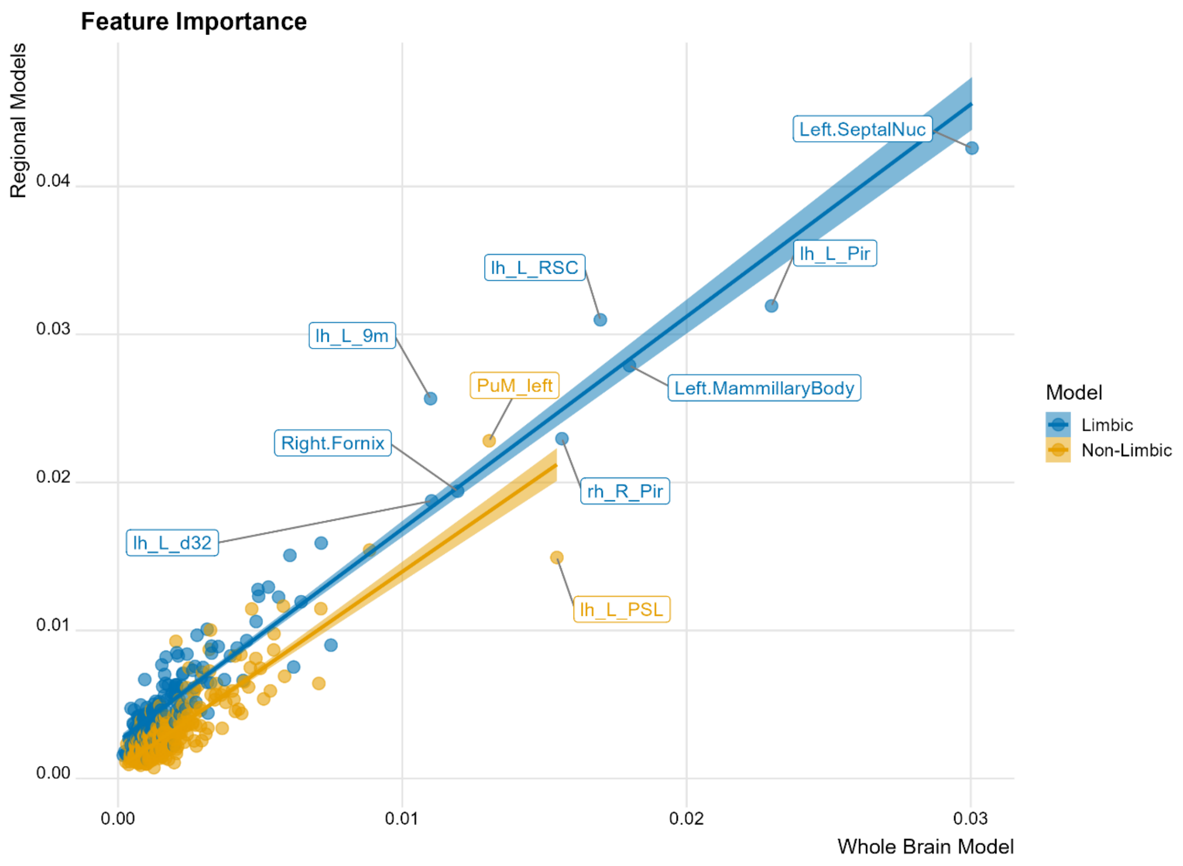

Figure S1. Comparisons of feature contributions between regionally constrained and whole brain models. Features contributing the most to the limbic and non-limbic models also contributed the most to the whole brain model. Regions contributing the most to the limbic model were the septal nuclei, the piriform cortex, subregions of the anterior and posterior cingulate cortex and the mammillary body. Regions contributing the most to the non-limbic model were the peri-Sylvian language area and the left medial pulvinar. Scatter plot depicts feature gains and regression lines with 95% confidence interval for limbic and non-limbic features between whole brain and regionally constrained models.

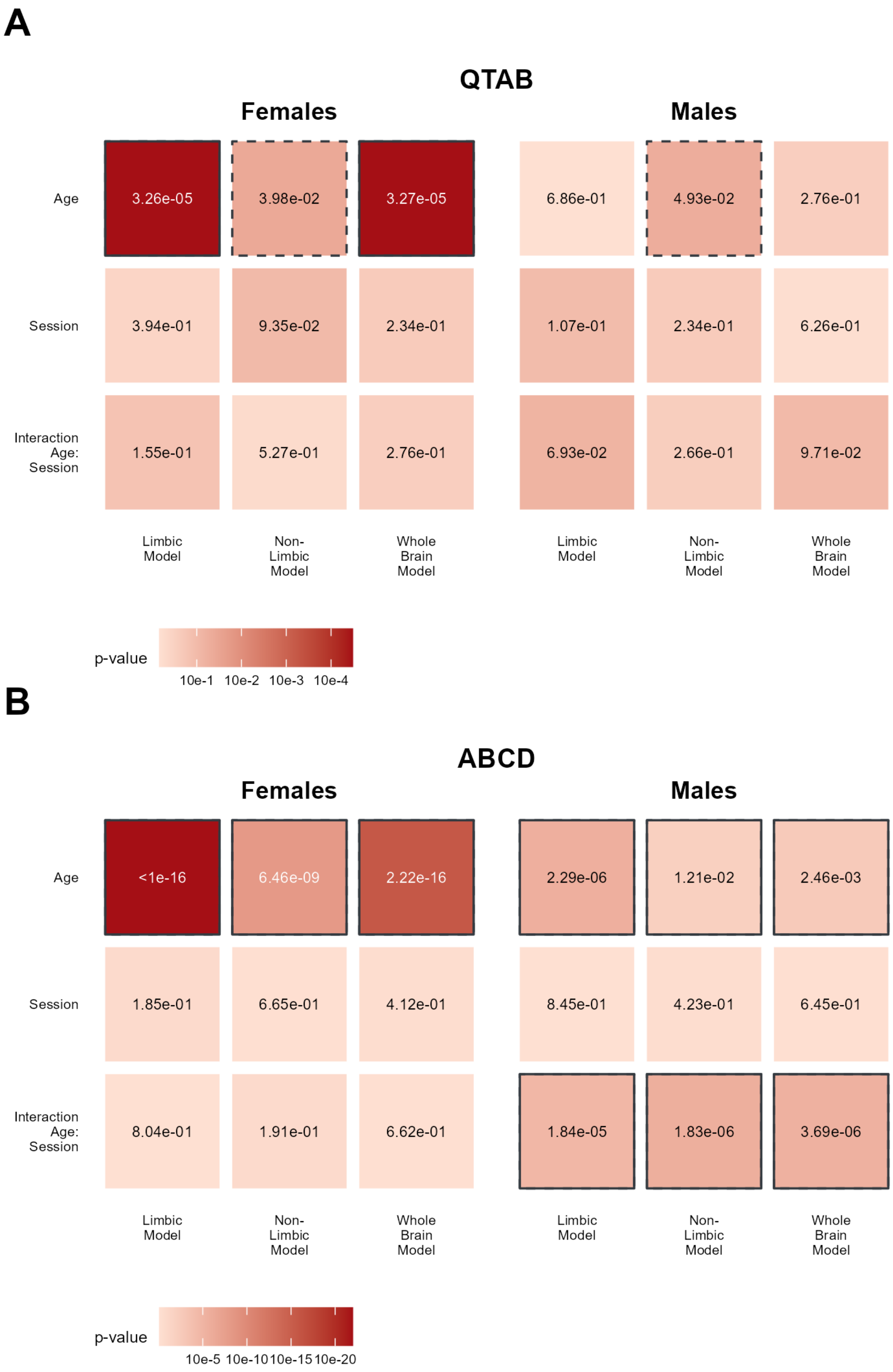
A
QTAB
Females
Males
Age
Session
Interaction Age: Session
3.26e-05
3.98e-02
3.27e-05
6.86e-01
4.93e-02
2.76e-01
3.94e-01
9.35e-02
2.34e-01
1.07e-01
2.34e-01
6.26e-01
1.55e-01
5.27e-01
2.76e-01
6.93e-02
2.66e-01
9.71e-02
Limbic Model
Non-Limbic Model
Whole Brain Model
Limbic Model
Non-Limbic Model
Whole Brain Model
p-value
10e-1
10e-2
10e-3
10e-4
B
ABCD
Females
Males
Age
Session
Interaction Age: Session
<1e-16
6.46e-09
2.22e-16
2.29e-06
1.21e-02
2.46e-03
1.85e-01
6.65e-01
4.12e-01
8.45e-01
4.23e-01
6.45e-01
8.04e-01
1.91e-01
6.62e-01
1.84e-05
1.83e-06
3.69e-06
Limbic Model
Non-Limbic Model
Whole Brain Model
Limbic Model
Non-Limbic Model
Whole Brain Model
p-value
10e-5
10e-10
10e-15
10e-20

Figure S2. Class probabilities are significantly associated with age, with stronger association in females compared to males. The maps depict the p-values associated with the F-statistics presented in Figure 1 of the main manuscript for each dataset, derived with Linear Mixed Effect (LME) models. Solid borders denote significance after correction for multiple comparisons ($p < .017$), dashed border denote nominally significant values that do not survive correction for multiple comparisons ($p < .05$)

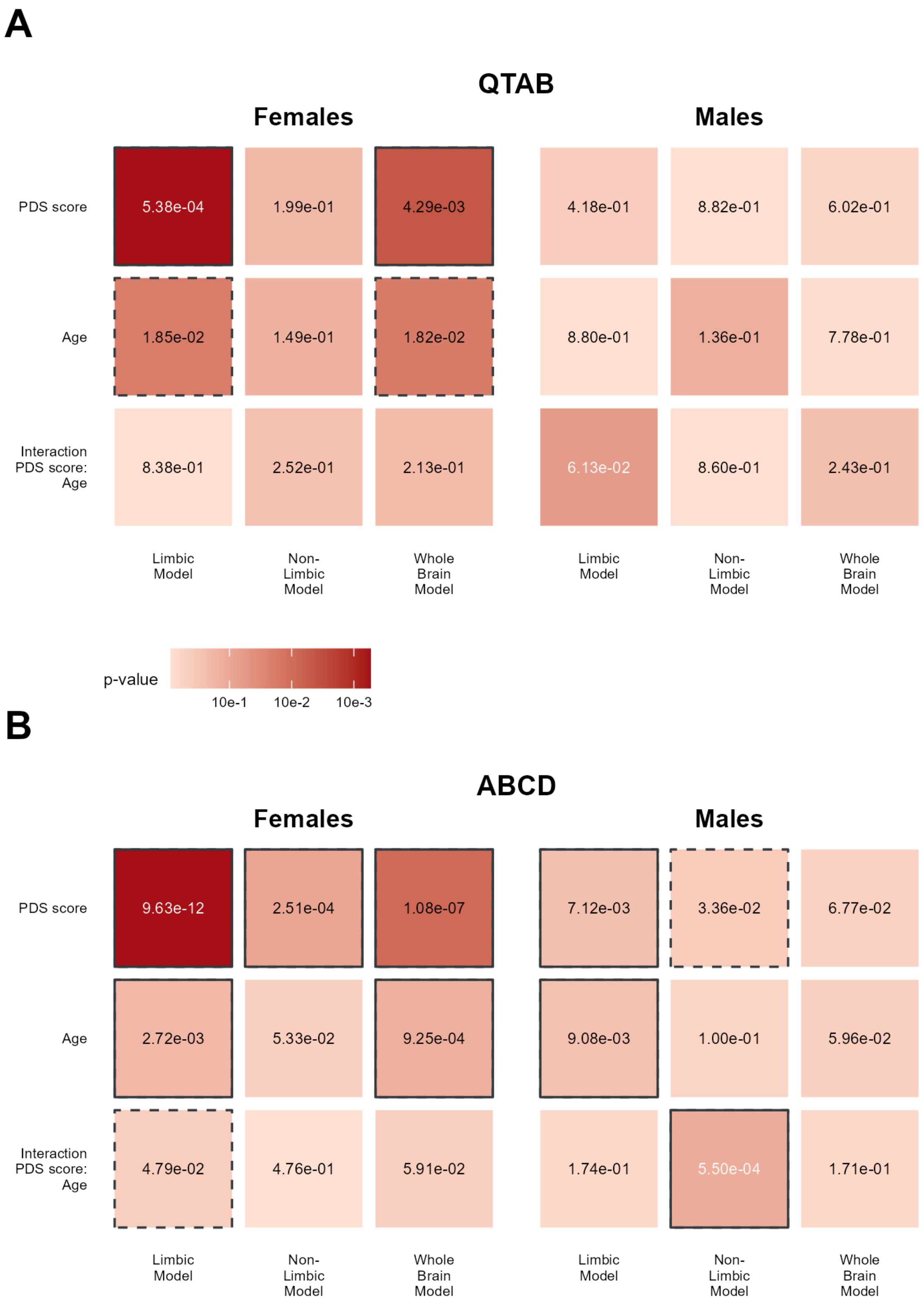
A
QTAB
Females
Males
PDS score
Age
Interaction PDS score: Age
5.38e-04
1.99e-01
4.29e-03
4.18e-01
8.82e-01
6.02e-01
1.85e-02
1.49e-01
1.82e-02
8.80e-01
1.36e-01
7.78e-01
8.38e-01
2.52e-01
2.13e-01
6.13e-02
8.60e-01
2.43e-01
Limbic Model
Non-Limbic Model
Whole Brain Model
p-value
10e-1
10e-2
10e-3
B
ABCD
Females
Males
PDS score
Age
Interaction PDS score: Age
9.63e-12
2.51e-04
1.08e-07
7.12e-03
3.36e-02
6.77e-02
2.72e-03
5.33e-02
9.25e-04
9.08e-03
1.00e-01
5.96e-02
4.79e-02
4.76e-01
5.91e-02
1.74e-01
5.50e-04
1.71e-01
Limbic Model
Non-Limbic Model
Whole Brain Model
p-value
10e-3
10e-6
10e-9

Figure S3. Class probabilities are associated with average PDS score with a pattern of strongest results in females and in the limbic system across datasets. The maps depict the p-values associated with the F-statistics presented in Figure 2 of the main manuscript for each dataset, derived with Linear Mixed Effect (LME) models. Solid borders denote significance after correction for multiple comparisons ($p < .017$), dashed borders denote nominally significant values that do not survive correction for multiple comparisons ($p < .05$).

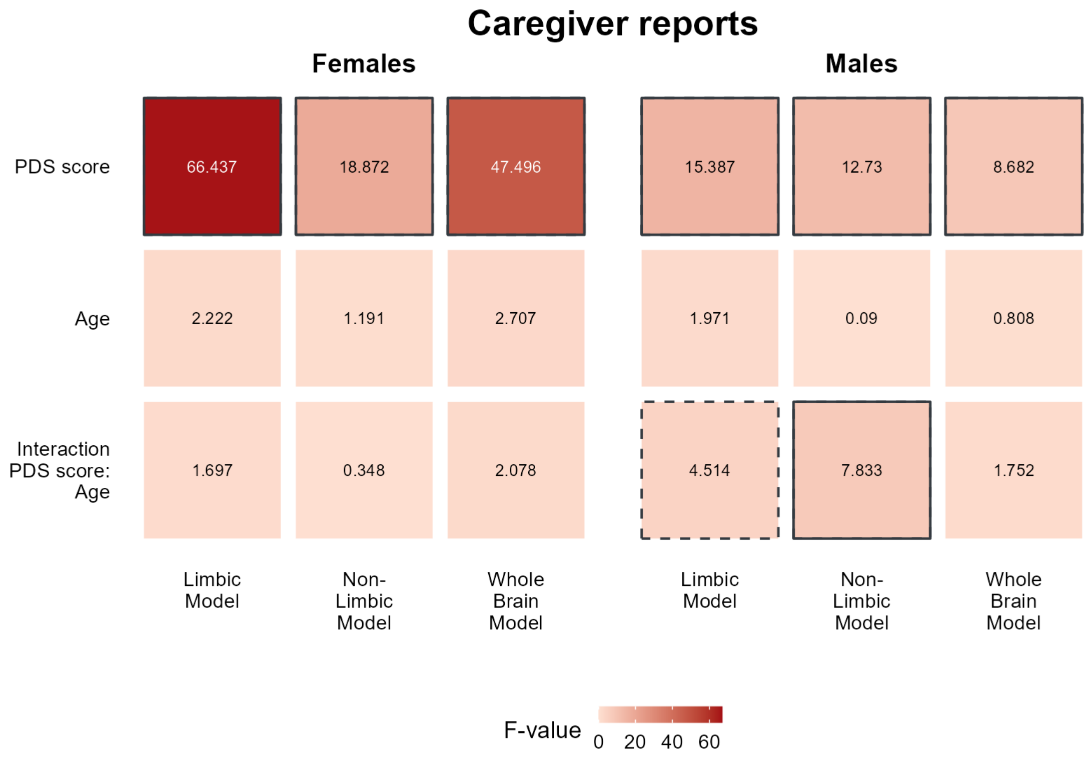

Figure S4. Caregiver's reports Pubertal Developmental Scale (PDS) score is associated with class probabilities in ABCD. In line with the findings obtained with self-reported PDS, limbic class probabilities showed strongest associations with PDS score with a pattern of stronger associations in females compared to males. Solid borders denote significance after correction for multiple comparisons ($p < .017$), dashed borders denote nominally significant values that do not survive correction for multiple comparisons ($p < .05$).

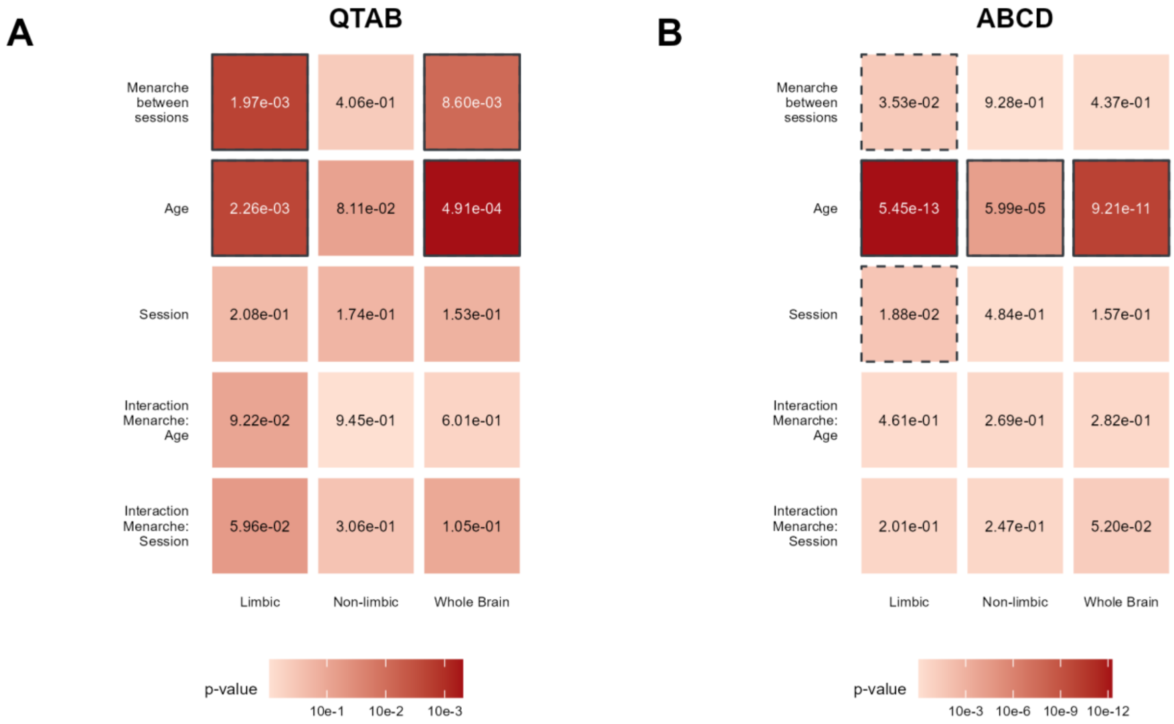

Figure S5. Limbic class probabilities are sensitive to menarche onset. The maps depict the p-values associated with the F-statistics presented in Figure 3 of the main manuscript for each dataset, derived with Linear Mixed Effect (LME) models. Solid borders denote significance after correction for multiple comparisons ($p < .017$), dashed borders denote nominally significant values that do not survive correction for multiple comparisons ($p < .05$).

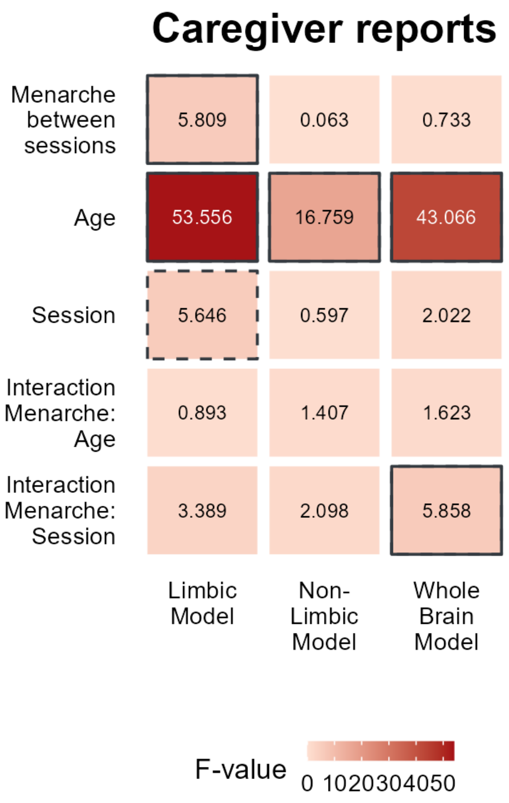

Figure S6. Menarche between sessions derived from caregiver's reports is associated with limbic class probabilities in ABCD. Solid borders denote significance after correction for multiple comparisons (p < .017), dashed borders denote nominally significant values that do not survive correction for multiple comparisons (p < .05).

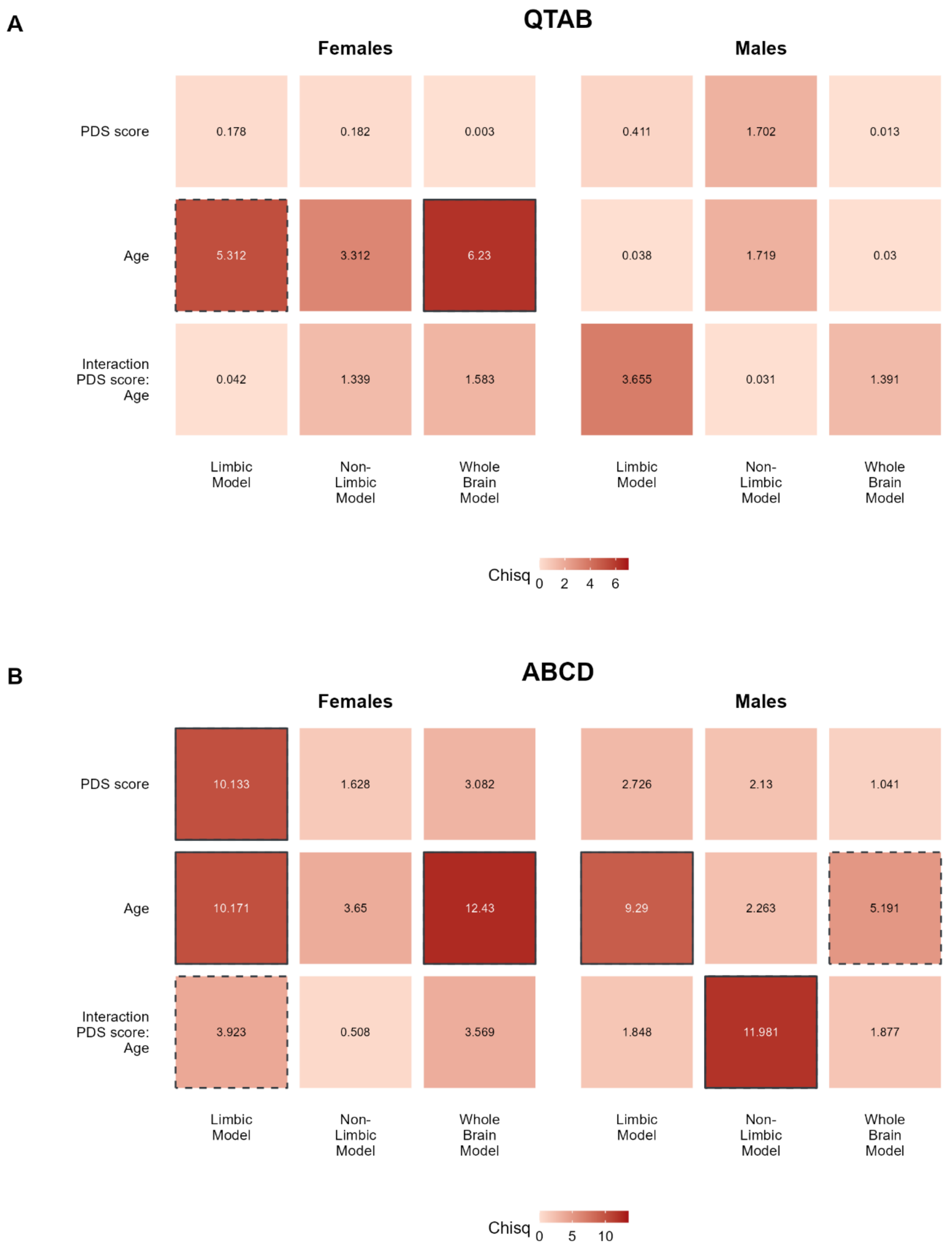

Figure S7. Relative contributions of age and PDS score to the associations with class probabilities, by ANOVA type-II. A) In QTAB, age explained most of the variance in the associations with class probabilities for both sexes. B) In ABCD, limbic class probabilities in females are significantly associated with PDS beyond age, while age has strongest effects for the other models and in males. Solid borders denote significance after correction for

multiple comparisons ($p < .017$), dashed borders denote nominally significant values that do not survive correction for multiple comparisons ($p < .05$).

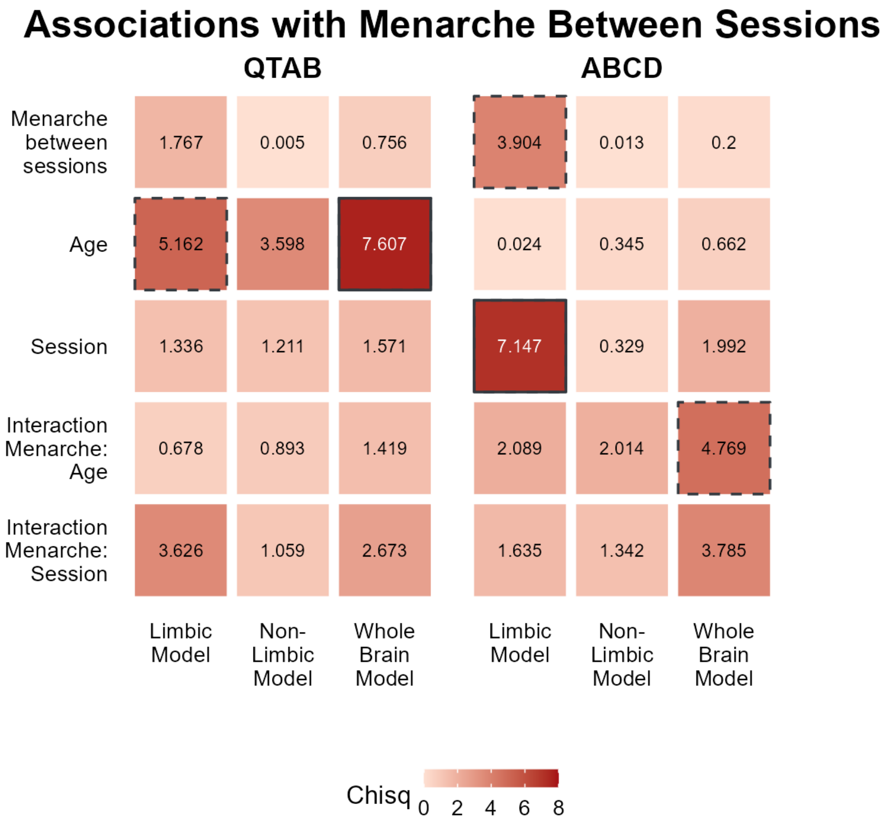

Figure S8. Relative contributions of age and menarche onset between sessions with ANOVA type-II. A) In QTAB, age explained most of the variance, while B) a significant association of menarche onset between sessions was found in ABCD. Solid borders denote significance after correction for multiple comparisons ($p < .017$), dashed borders denote nominally significant values that do not survive correction for multiple comparisons ($p < .05$).

A

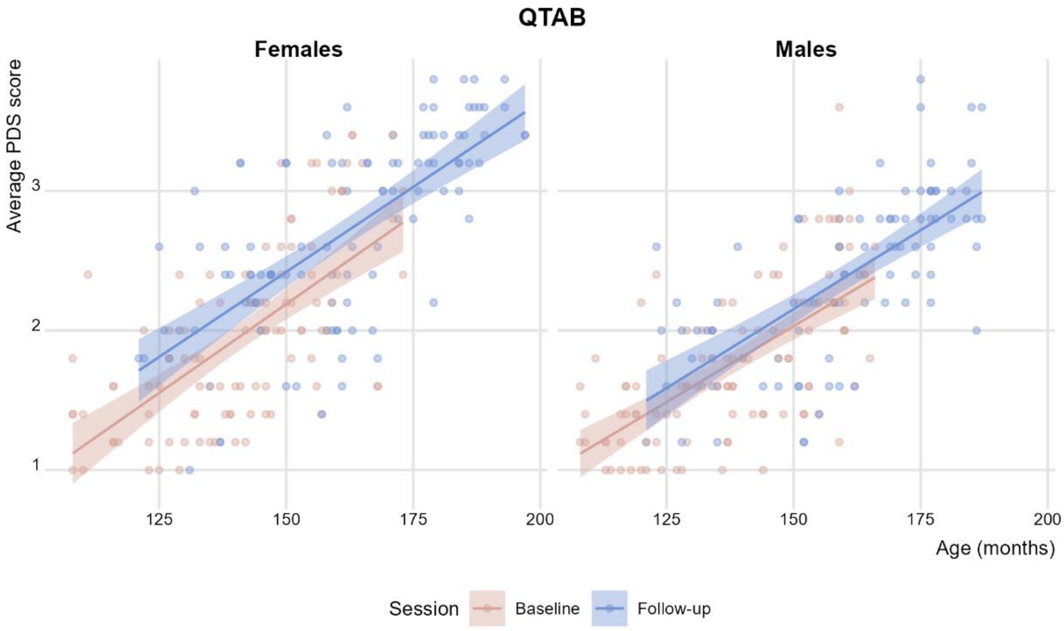

B

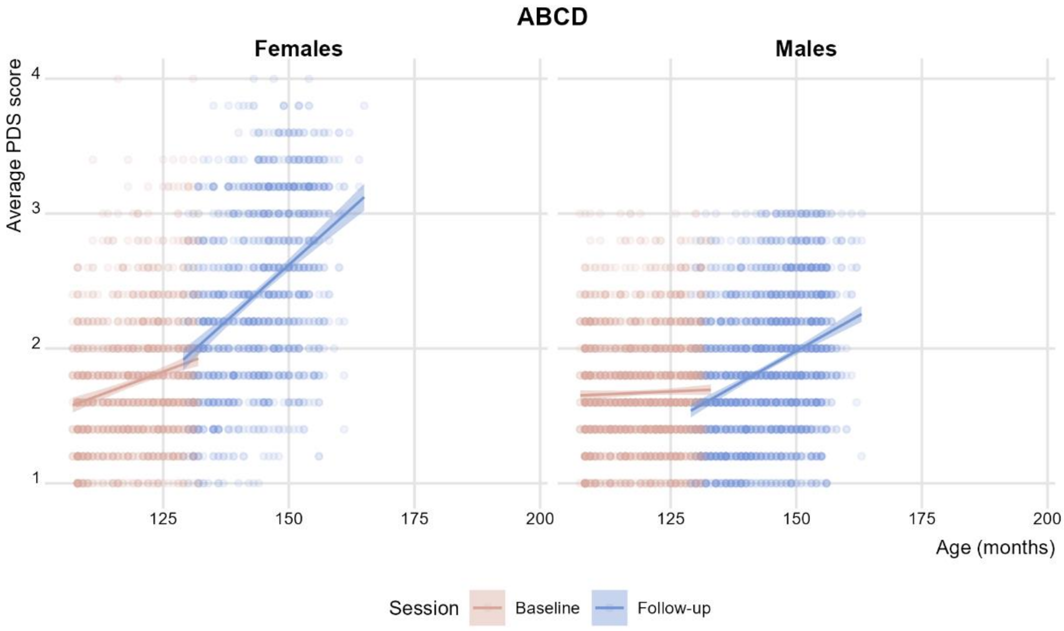

Figure S9. Correlation between PDS score and age. A) In QTAB, strong significant correlations were found at each session in both females and males. No significant difference in correlations between sessions were found. B) In ABCD, females showed a significant correlation between age and PDS score for both sessions, while males showed a significant correlation only at follow-up. Moreover, a significant difference between correlation at baseline

and follow-up was found in each sex. Regression line with 95% confidence interval between data points at baseline and follow-up are shown.

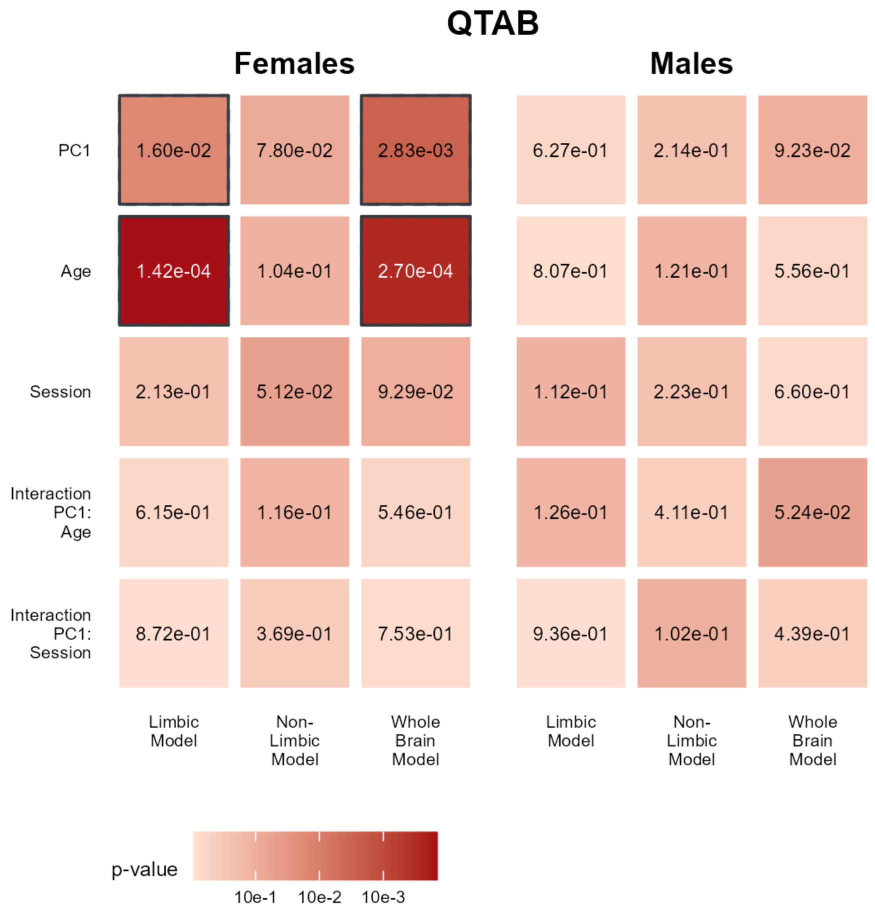

Figure S10. Class probabilities are associated with mental health in a sex-specific manner. The maps depict the p-values associated with the F-statistics presented in Figure 4 of the main manuscript for each dataset, derived from Linear Mixed Effect (LME) models. Solid borders denote significance after correction for multiple comparisons (p < .017).

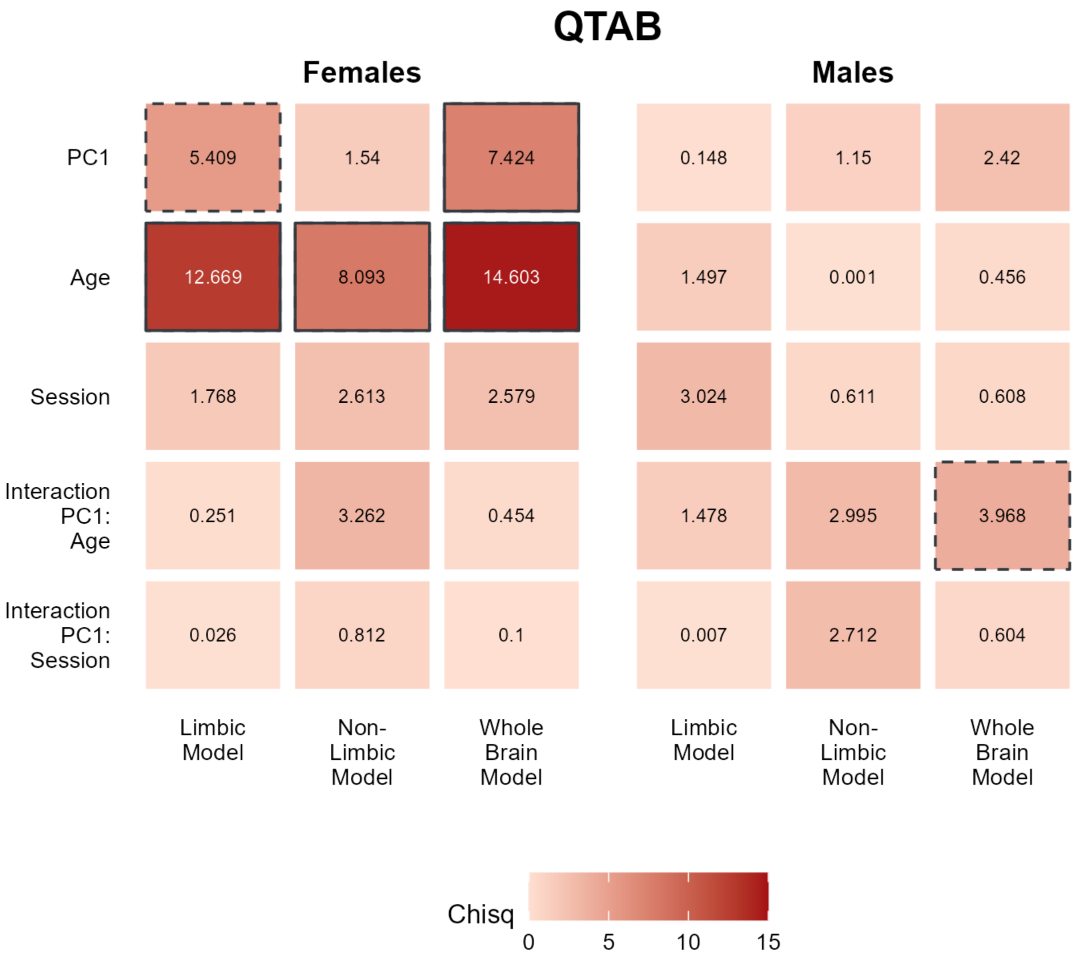

Figure S11. Class probabilities are associated with both mental health status beyond age in a sex-specific manner. Anova type-II results showed significant association of whole brain and limbic class probabilities with mental health score beyond age in females, while no significant effect was found in males. After correction for multiple comparisons only the whole brain effect survived. Solid borders denote significance after correction for multiple comparisons ($p < .017$), dashed borders denote nominally significant values that do not survive correction for multiple comparisons ($p < .05$).

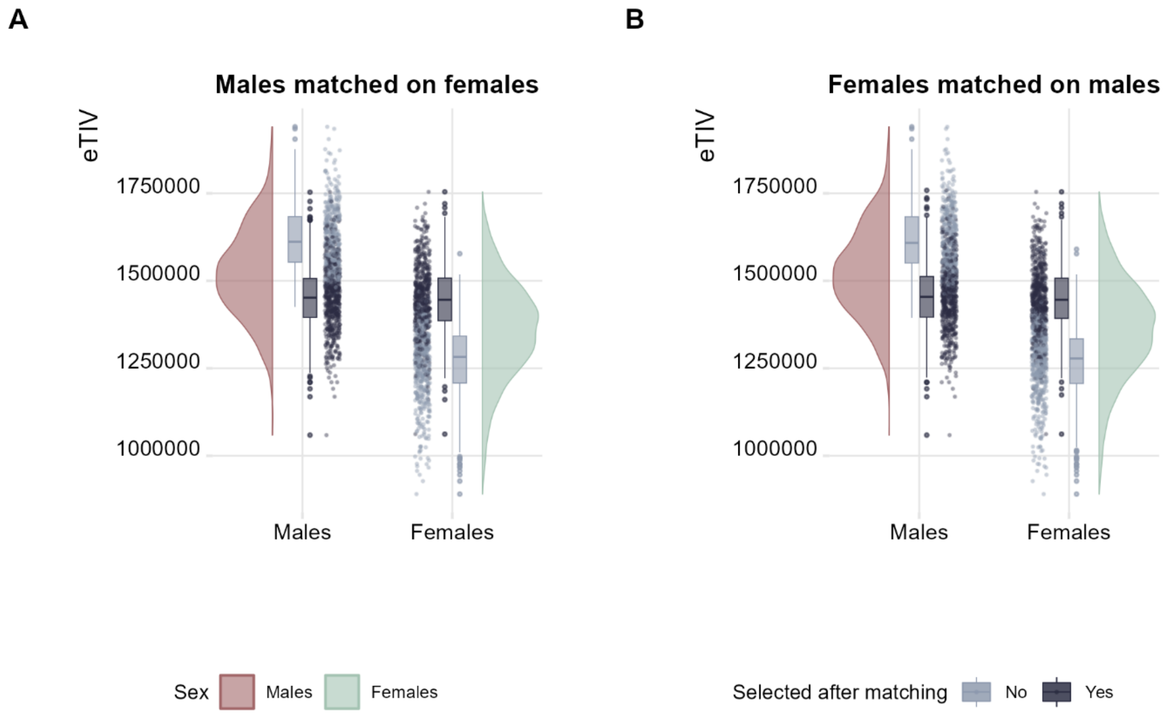

Figure S12. Matching procedure using each of the two sexes as reference leads to comparable Estimated Total Intracranial Volume (eTIV) distribution. A) eTIV distribution when females are taken as reference, corresponding to the training set used in the main paper. B) Supplementary analysis using males as reference leads to similar distribution. The whiskers in the boxplot extend from the hinge to the largest/smallest value no further than 1.5 * IQR from the hinge.

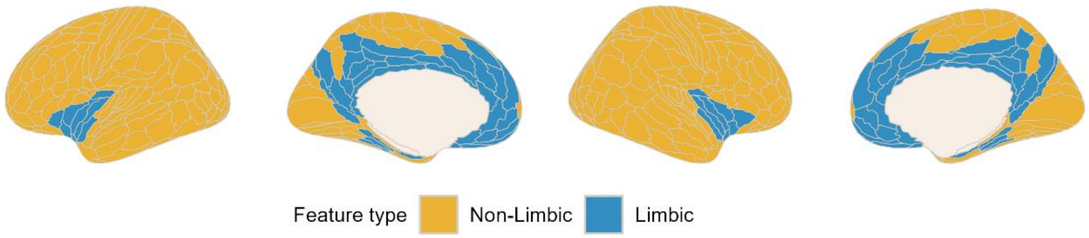

Figure S13. Cortical distribution of limbic and non-limbic features.

Supplementary Tables

	Tot	Females	Males
**Total Sample**			
N	2054	1086	968
age	179 ± 44.5	181 ± 45.0	178 ± 43.9
eTIV	1436981 ± 154645.9	1361084 ± 136872.0	1522131 ± 126595.0
**Selected**	**Tot**	**Females**	**Males**
N	1132	566	566
age	174 ± 44.6	174 ± 44.5	174 ± 44.7
eTIV	1449042 ± 98151.0	1447216 ± 99663.0	1450869 ± 96670.0
**Excluded**	**Tot**	**Females**	**Males**
N	922	520	402
age	187 ± 43.4	188 ± 44.3	184 ± 42.0
eTIV	1422173 ± 202685.0	1267332 ± 107026.0	1622465 ± 90666.0

Table T1. Training sample characteristics before and after the matching procedure for the selected and excluded participants. The eTIV distributions support that the matching procedure successfully removed the eTIV difference between males and females, while still staying within the bounds of a reasonably representative sample. Mean ± standard deviation (SD) of age (expressed in months) and eTIV.

				Baseline		Follow-up	
				**Females**	**Males**	**Females**	**Males**
QTAB							
	Age			137 ± 16.87	136 ± 15.79	158 ± 19.14	155 ± 17.54
	N						
		Tot		192	200	152	138
		PDS		185	179	145	119
		Menarche		110	-	110	-
			No menarche	64	-	64	-
			Between sessions	46	-	46	-
		Mental Health		192	200	152	138
ABCD							
	Age			119 ± 7.41	119 ± 7.49	143 ± 7.78	144 ± 7.75
	N						
		Tot		3629	4121	3478	4070
		PDS		1173	1846	1159	1841
		Menarche		1091	-	1091	-
			No menarche	677	-	677	-
			Between sessions	414	-	414	-

Table T2. Included participants for each association analysis in the two longitudinal samples. Age is expressed in months and reported as mean ± standard deviation (SD)

Table T3. List of limbic structures with the correspondent feature in Freesurfer

Structure	FreeSurfer Feature	Segmentation
Anterior Cingulate Cortex	33pr	Glasser atlas
	p24pr	
	a24pr	
	p24	
	a24	
	p32pr	
	a32pr	
	d32	
	p32	
	s32	
	8BM	
	9m	
	10v	
	10r	
	25	
Orbitofrontal Cortex	OFC	
	pOFC	
Insula	MI	
	AVI	
	AAIC	
	Ig	
	PI	
	PoI1	

Structure	FreeSurfer Feature	Segmentation
	PoI2	
Piriform Cortex	Pir	
Entorhinal Cortex	EC	
Parahippocampal Area	PHA1	
	PHA2	
	PHA3	
Posterior Cingulate Cortex	DVT	
	ProS	
	POS1	
	POS2	
	RSC	
	v23ab	
	d23ab	
	31pv	
	31pd	
	31a	
	23d	
	23c	
	PCV	
Hippocampus	Hippocampal_tail	Hippocampal Subfields
	subiculum-body	
	CA1-body	
	subiculum-head	
	hippocampal-fissure	
	presubiculum-head	
	CA1-head	
	presubiculum-body	

Structure	FreeSurfer Feature	Segmentation
	parasubiculum	
	molecular_layer_HP-head	
	molecular_layer_HP-body	
	GC-ML-DG-head	
	CA3-body	
	GC-ML-DG-body	
	CA4-head	
	CA4-body	
	fimbria	
	CA3-head	
	HATA	
Amygdala	Lateral-nucleus	Nuclei of Amygdala
	Basal-nucleus	
	Accessory-Basal-nucleus	
	Anterior-amygdaloid-area-AAA	
	Central-nucleus	
	Medial-nucleus	
	Cortical-nucleus	
	Corticoamygdaloid-transitio	
	Paralaminar-nucleus	
Anterior and Dorsomedial Thalamic Nuclei	AV	Thalamic Nuclei
	LD	
	MDl	
	MDm	
Nucleus Accumbens	Nucleus-Accumbens	ScLimbic
Hypothalamus	HypoThal-noMB	
Fornix	Fornix	

Structure	FreeSurfer Feature	Segmentation
Mammillary Body	MammillaryBody	
Forebrain	Basal-Forebrain	
Septal Nuclei	SeptalNuc	
