# Supplementary material for: Longitudinal development of sex differences in the limbic system is associated with age, puberty and mental health
Source: Commun Biol. 2025 Nov 5;8:1524. doi: 10.1038/s42003-025-08866-3 (PMC12589509; doi:10.1038/s42003-025-08866-3)
Supplement: Supplementary file 3 — Reporting Summary [file 42003_2025_8866_MOESM3_ESM.pdf]

nature portfolio

Corresponding author(s): Gloria Matte Bon, Tobias Kaufmann

Last updated by author(s): Aug 5, 2025

Reporting Summary

Nature Portfolio wishes to improve the reproducibility of the work that we publish. This form provides structure for consistency and transparency in reporting. For further information on Nature Portfolio policies, see our Editorial Policies and the Editorial Policy Checklist.

Statistics

For all statistical analyses, confirm that the following items are present in the figure legend, table legend, main text, or Methods section.

n/a	Confirmed	
☐	☒	The exact sample size (n) for each experimental group/condition, given as a discrete number and unit of measurement
☐	☒	A statement on whether measurements were taken from distinct samples or whether the same sample was measured repeatedly
☐	☒	The statistical test(s) used AND whether they are one- or two-sided<br>*Only common tests should be described solely by name; describe more complex techniques in the Methods section.*
☐	☒	A description of all covariates tested
☐	☒	A description of any assumptions or corrections, such as tests of normality and adjustment for multiple comparisons
☐	☒	A full description of the statistical parameters including central tendency (e.g. means) or other basic estimates (e.g. regression coefficient) AND variation (e.g. standard deviation) or associated estimates of uncertainty (e.g. confidence intervals)
☐	☒	For null hypothesis testing, the test statistic (e.g. F, t, r) with confidence intervals, effect sizes, degrees of freedom and P value noted<br>*Give P values as exact values whenever suitable.*
☒	☐	For Bayesian analysis, information on the choice of priors and Markov chain Monte Carlo settings
☒	☐	For hierarchical and complex designs, identification of the appropriate level for tests and full reporting of outcomes
☐	☒	Estimates of effect sizes (e.g. Cohen's d, Pearson's r), indicating how they were calculated

Our web collection on statistics for biologists contains articles on many of the points above.

Software and code

Policy information about availability of computer code

Data collection	No data collection was performed by the authors. This is an analysis of publicly available data (i.e., PNC, HCP-D, QTAB, ABCD).
Data analysis	Magnetic resonance imaging (MRI) data were processed with FreeSurfer (version 7.1.1).<br>All statistical analyses were performed in R (version 4.3.1).<br>Code will be made publicly available at https://github.com/gloriamatte upon publication.

For manuscripts utilizing custom algorithms or software that are central to the research but not yet described in published literature, software must be made available to editors and reviewers. We strongly encourage code deposition in a community repository (e.g. GitHub). See the Nature Portfolio guidelines for submitting code & software for further information.

Data

Policy information about availability of data

All manuscripts must include a data availability statement. This statement should provide the following information, where applicable:

- Accession codes, unique identifiers, or web links for publicly available datasets
- A description of any restrictions on data availability
- For clinical datasets or third party data, please ensure that the statement adheres to our policy

Data used in this study was accessed under data use agreements with the respective study cohorts (PNC, HCP-D, ABCD, QTAB). All raw data is available via dedicated data use agreements with the data providers.

Research involving human participants, their data, or biological material

Policy information about studies with human participants or human data. See also policy information about sex, gender (identity/presentation), and sexual orientation and race, ethnicity and racism.

Reporting on sex and gender	This study investigates a multivariate brain-derived measure of sex as main variable of interest. Sex was defined as biological sex assigned at birth. Statistical biological associations were reported separately in males and females, as opposite directions of the effects are expected for each sex.
Reporting on race, ethnicity, or other socially relevant groupings	n.a
Population characteristics	This study includes data from four publicly available datasets, including healthy participants covering an age range between 8 and 22 years old. For the training set we used sex and age-matched individuals from the PNC and HCP-D, for a total of N = 1132 (50% females; PNC = 768 of which 48% females; HCP-D = 364 of which 53% females, age range: 8-22 years old). QTAB and ABCD were used as independent test samples. The QTAB dataset includes adolescent twins (N at baseline = 392, 49% females, age at baseline: 9-14 years), scanned twice with an interval of 13 to 30 months (mean interval: 20 months). For the ABCD data we used data release 5.0, including two MRI scans collected 2 years apart, across multiple research study sites (N at baseline = 7792, 47% females, age at baseline: 9-11 years).
Recruitment	Detailed information can be retrieved from the original manuscripts describing the respective studies:<br>PNC: see Satterwhaite et al. (2016), doi: 10.1016/j.neuroimage.2013.07.064.<br>HCP-D: see Sommerville et al. (2018), doi: 10.1016/j.neuroimage.2018.08.050.<br>QTAB: Strike et al. (2023), doi: 10.1038/s41597-023-02038-w.<br>ABCD: Casey et al. (2018), doi: 10.1016/j.dcn.2018.03.001.
Ethics oversight	PNC: institutional review board (IRB) of University of Pennsylvania, Children's Hospital of Philadelphia<br>HCP-D: IRBs of the participating sites: Harvard University, Oxford University, University of California Los Angeles, University of Minnesota, Washington University<br>QTAB: Children's Health Queensland Human Research Ethics Committee (HREC) (reference HREC/16/QRCH/270) University of Queensland HREC (reference 2016001784)<br>ABCD: IRB of the local site or by local IRB reliance agreements with the central IRB of the University of California-San Diego

Note that full information on the approval of the study protocol must also be provided in the manuscript.

Field-specific reporting

Please select the one below that is the best fit for your research. If you are not sure, read the appropriate sections before making your selection.

☒ Life sciences ☐ Behavioural & social sciences ☐ Ecological, evolutionary & environmental sciences

For a reference copy of the document with all sections, see nature.com/documents/nr-reporting-summary-flat.pdf

Life sciences study design

All studies must disclose on these points even when the disclosure is negative.

Sample size	Sample size was based on data availability, including the maximum number of MRI data available for each sample.
Data exclusions	For MRI data, participants with image quality below 3SD from the mean were excluded. For association analyses, participants with missing data were excluded from the analyses.
Replication	Model training was performed in PNC and HCP-D data. Model validation was carried out in independent data from the ABCD and QTAB. All association analyses were replicated in multiple samples (e.g., age associations in training and both test samples indipendently), with the exception of mental health associations that were carried out only in QTAB due to the limited age range in the ABCD.
Randomization	Randomization does not apply for this study.
Blinding	Blinding does not apply for this study.

Reporting for specific materials, systems and methods

We require information from authors about some types of materials, experimental systems and methods used in many studies. Here, indicate whether each material, system or method listed is relevant to your study. If you are not sure if a list item applies to your research, read the appropriate section before selecting a response.

Materials & experimental systems

n/a	Involved in the study
☒	☐ Antibodies
☒	☐ Eukaryotic cell lines
☒	☐ Palaeontology and archaeology
☒	☐ Animals and other organisms
☒	☐ Clinical data
☒	☐ Dual use research of concern
☒	☐ Plants

Methods

n/a	Involved in the study
☒	☐ ChIP-seq
☒	☐ Flow cytometry
☐	☒ MRI-based neuroimaging

Plants

Seed stocks: NOT INVOLVED

Novel plant genotypes: NOT INVOLVED

Authentication: NOT INVOLVED

Magnetic resonance imaging

Experimental design

Design type: Structural MRI

Design specifications: n.a.

Behavioral performance measures: n.a.

Acquisition

Imaging type(s): Structural T1 weighted

Field strength: 3T

Sequence & imaging parameters: Detailed information can be retrieved from the original manuscripts describing the respective studies:
PNC: see Satterwhaite et al. (2016), doi: 10.1016/j.neuroimage.2013.07.064.
HCP-D: see Sommerville et al. (2018), doi: 10.1016/j.neuroimage.2018.08.050.
QTAB: Strike et al. (2023), doi: 10.1038/s41597-023-02038-w.
ABCD: Casey et al. (2018), doi: 10.1016/j.dcn.2018.03.001.

Area of acquisition: Whole brain

Diffusion MRI: ☐ Used ☒ Not used

Preprocessing

Preprocessing software: Raw data were preprocessed with FreeSurfer (version 7.1.1). A combination of different segmentations methods were applied, including recon-all, multimodal parcellation of the cerebral cortex, segmentation of subcortical limbic structures, subfield segmentation of the hippocampus, amygdala and thalamus.

Normalization: n.a

Normalization template: n.a

Noise and artifact removal: n.a

Volume censoring: n.a

Statistical modeling & inference

Model type and settings	We used multivariate binary classification implemented with xgboost in R to derive measures of brain sex from brain volumes. To test biological associations we used linear models for cross-sectional data and linear mixed effect models for longitudinal datasets. For mental health associations we derived a mental health score using principal component analysis for dimensionality reduction.
Effect(s) tested	All associations analyses accounted for age, image quality, session (when applicable) and site (when applicable). ANOVA type I and type II were applied to the output of Linear Mixed Effect models to obtain F-statistics and p-values.

Specify type of analysis: ☐ Whole brain ☒ ROI-based ☐ Both

Anatomical location(s)	Glasser Atlas, ScLimbic, Hippocampal subfield , Nuclei of Amygdala, Thalamic Nuclei, subcortical FreeSurfer
Statistic type for inference (See Eklund et al. 2016)	-
Correction	Bonferroni

Models & analysis

n/a	Involved in the study
☒	☐ Functional and/or effective connectivity
☒	☐ Graph analysis
☐	☒ Multivariate modeling or predictive analysis

Multivariate modeling and predictive analysis	Different combinations of brain volumes corrected for total brain size were used as features for machine learning models. We performed a nested 5-fold cross validation, with the learning rate η=0.01 and the initial number of rounds set to 1000. The prediction error in the inner loop was assessed for each iteration and used to determine the optimal number of iterations to train the final models on the entire set of data. To assess each model performance we computed the area under the receiving operating characteristic curves (AUC-ROC curves).
